# Right Ventricular Implantation of an Aveir™ AR Device for Bradycardia in a 90-Year-Old Woman with Atrial Fibrillation and Small Right Ventricular Dimensions

**DOI:** 10.19102/icrm.2026.17061

**Published:** 2026-06-15

**Authors:** Ali Sovari, Leili Pourafkari, Jeong Hwan J. Kim

**Affiliations:** 1 Department of Cardiology, St. John’s Regional Medical Center, Oxnard, CA, USA; 2 Department of Cardiovascular Disease, Los Robles Regional Medical Center, Thousand Oaks, CA, USA; 3 Department of Anesthesiology, Jacobs School of Medicine and Biomedical Sciences, Buffalo, NY, USA

**Keywords:** Leadless pacemaker, leadless pacemaker extraction, symptomatic bradycardia

## Abstract

We report a case of implanting an Aveir™ AR (atrial) leadless pacemaker (Abbott, Chicago, IL, USA) in the right ventricle (RV) of a 90-year-old woman with permanent atrial fibrillation with pauses. The patient initially received an Aveir™ VR (ventricular) device for rate support. During implantation, elevated pacing thresholds and tricuspid valve (TV) interaction were noted, which improved with repositioning. The patient subsequently presented with recurrent loss of capture secondary to elevated thresholds. Device interrogation and imaging suggested that the Aveir™ VR device was suboptimal due to the patient’s small RV cavity and interaction with TV. To address this, the pacemaker was extracted, and an Aveir™ AR device—featuring a smaller profile—was successfully implanted in the RV, achieving stable fixation. To the best of our knowledge, this is the first reported case of off-label RV implantation of an Aveir™ AR device. This case illustrates the versatility of modular leadless pacing systems and underscores the importance of tailoring device selection to individual cardiac anatomy, particularly TV, in elderly patients with small RV dimensions.

## Introduction

Leadless pacemakers (LPMs) offer significant advantages over traditional transvenous pacing systems by eliminating intravascular leads and subcutaneous pockets, thereby reducing the risk of infection, lead dislodgement or fracture, venous thrombosis, and tricuspid valve (TV) dysfunction.^1^ The Aveir™ VR system (Abbott, Chicago, IL, USA) is an LPM that incorporates active helix fixation, pre-deployment electrical mapping, and retrievability—features^2^ that are particularly advantageous for patients with atrial fibrillation (AF) and bradycardia who require ventricular-only pacing. However, anatomical factors, such as small right ventricular (RV) dimensions, can present technical challenges during LPM implantation,^3^ including difficulty achieving optimal pacing thresholds and avoiding mechanical interaction with cardiac structures, particularly TV.

We present, to the best of our knowledge, the first reported case of RV implantation of an Aveir™ AR (atrial) device, used off-label to manage recurrent loss of capture with an initially implanted Aveir™ VR, attributed to small RV dimensions and resultant interference with TV.

This case highlights the adaptability of modular leadless pacing systems and the importance of tailoring device selection to individual patient anatomy.

## Case presentation

A 90-year-old woman with persistent AF and symptomatic bradycardia was referred for permanent pacemaker implantation. Transthoracic echocardiogram showed normal left ventricular ejection fraction, biatrial enlargement, and severe functional tricuspid regurgitation. Given her chronic AF and absence of sinus rhythm, a single-chamber ventricular pacing system was selected. Considering the patient’s advanced age, tricuspid regurgitation, and limited pectoral tissue, she was deemed high risk for complications from a transvenous device, and an LPM was chosen. An Aveir™ VR device was implanted via right femoral venous access into the RV septum.

Intraprocedural testing showed transiently elevated capture thresholds, although sensing and impedance were acceptable. Intracardiac echocardiography (ICE) revealed subtle interaction with the TV. This was initially mitigated by repositioning the device more apically, where improvement in capture thresholds was observed. The patient was discharged with stable thresholds.

**Figure 1:**
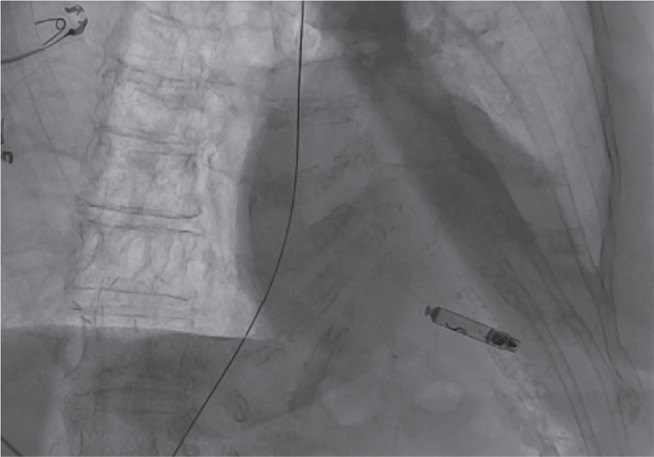
Original Aveir™ VR device interacting with the tricuspid apparatus.

Two weeks later, the patient presented with generalized weakness. Device interrogation revealed loss of capture and an elevated threshold of 2.25 mV, with a projected longevity of 3.1 years. Given her presentation, an extensive preoperative discussion was made, including the risks, benefits, and the possibility of off-label use of LPMs, with the patient and her surrogate decision-maker. Micro-dislodgment was suspected, and LPM revision was pursued. Repeated repositioning attempts were not successful due to the already apical position of the LPM and yielded no improvement in thresholds. Imaging and intraprocedural assessment revealed a small RV cavity and suboptimal positioning. Intraprocedural fluoroscopy demonstrated that the Aveir™ VR device was interacting with the TV apparatus and exhibited suboptimal stability at the initial implantation site **(Video 1, Figure 1)**.

**Figure 2:**
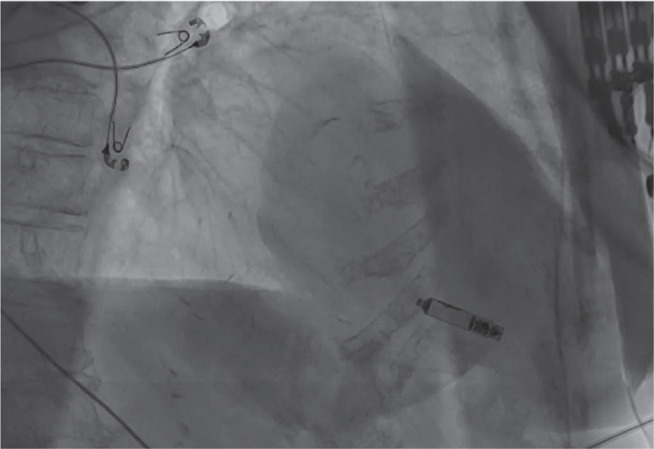
Final result with the stable Aveir™ AR device in the inferior septum of the right ventricle.

Accordingly, the previously implanted Aveir™ VR was first extracted **(Video 2)**. As a novel solution to address the anatomical constraints, an Aveir™ AR device—featuring a smaller profile (32.2 mm in length, 6.1 mm in diameter)—was selected for its reduced risk of TV interference and implanted in the RV apex. Although designed for atrial use, the Aveir™ AR shares an active helix fixation and pre-deployment mapping system very similar to that of the VR device. The AR device achieved excellent pacing parameters, with no mechanical interaction with the TV observed **(Video 3, Figure 2)**, and the patient was discharged without complications.

At 1- and 2-month follow-ups, the patient remained asymptomatic with stable pacing thresholds and appropriate device function. Device interrogation 2 months after implantation showed an RV threshold of 0.5 mV at a pulse width of 0.4 ms, a sensitivity of 2 mV, a lead impedance of 400 Ω, and a projected longevity of 9.6 years.

## Discussion

**Figure 3:**
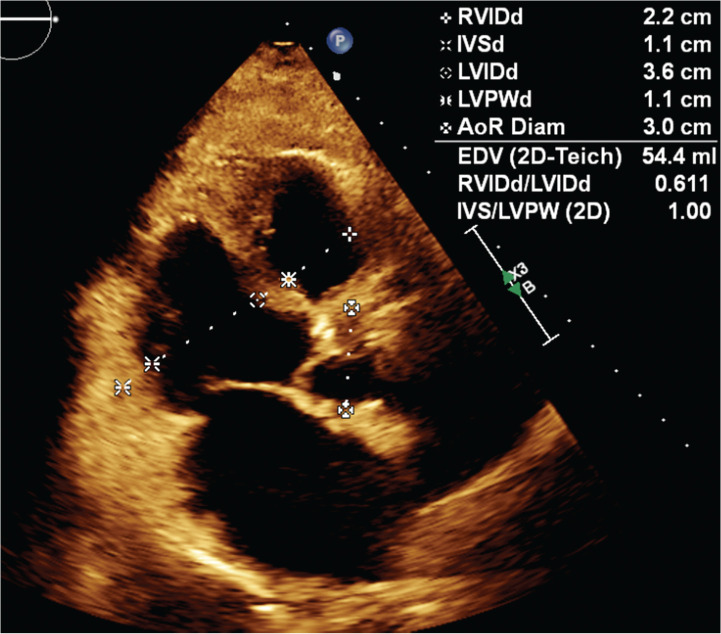
Preoperative transthoracic echocardiogram assessment with left and right ventricular dimensions. *Abbreviations:* AoR Diam, aortic root diameter; EDV, end-diastolic volume; IVS, interventricular septum; IVSd, interventricular septal thickness in diastole; LVIDd, left ventricular internal diameter in diastole; LVPW, left ventricular posterior wall; LVPWd, left ventricular posterior wall thickness in diastole; RVIDd, right ventricular internal diameter in diastole.

Leadless pacing technology continues to evolve, offering solutions that avoid the complications inherent in transvenous systems. The Aveir™ VR system was designed with retrievability and intracardiac mapping to enhance safety and performance, yet even this sophisticated platform may be limited by patient-specific anatomical challenges. Anatomical variations of the right heart, including TV abnormalities and altered chamber geometry, are recognized challenges during LPM implantation, as they can limit catheter support and complicate device fixation.^4^ In our case, a small RV cavity associated with TV interaction created similar technical difficulties, ultimately necessitating an alternative device choice **(Figure 3)**. Preoperative computed tomography (CT) in selected cases may aid in the decision-making process by quantifying the ventricular dimensions; however, the usefulness is subject to further investigation as the interaction between the LPM delivery system and RV is difficult to foresee solely based on the anatomical measurements. In addition, the cost-effectiveness of CT screening is in question as the incidence or frequency of a small RV in the adult population has not been well studied; it appears to be rare and is currently described only in isolated case reports.

In this case, repeated loss of capture following correct implantation and repositioning of the Aveir™ VR was likely due to a small RV cavity as well as interaction with TV and associated challenges in device anchoring and myocardial engagement. The observed TV interference further limited the effective placement zone. These limitations prompted the innovative use of an Aveir™ AR device, which is smaller and more suitable for the patient’s anatomy. The Aveir™ VR measures 38 mm in length and 6.5 mm in diameter, while the Aveir™ AR measures 32.2 mm in length and 6.1 mm in diameter.^5^ The reduced size and mass of the AR device allowed for more stable placement without tricuspid interaction. A minor difference between Aveir™ AR and VR is that Aveir™ AR has a dual-helix nose with an inactive outer helix for primary mechanical fixation and a recessed inner helix as a pacing electrode and secondary fixation, whereas Aveir™ VR has a single fixation helix. The Aveir™ AR shares the VR’s helix fixation design and mapping capabilities; in addition, it has the second fixation helix designed for more stability in a less-trabeculated chamber, making it a reasonable substitute from a technical standpoint despite its off-label use.

An alternative approach in this case could have included the use of the Micra™ pacemaker (Medtronic, Minneapolis, MN, USA), which also has a compact profile and uses passive tines for fixation. However, if Micra™ implantation results in TV interaction being detected after release, the patient would require LPM retrieval with a passive fixation mechanism.

Repeated repositioning or retrieval of leadless devices carries a small but clinically meaningful risk of cardiac perforation, pericardial effusion, and tamponade. Contemporary series and reviews have highlighted that, although overall complication rates are low, perforation events with leadless systems may be more severe than with traditional transvenous leads, underscoring the importance of minimizing the number of deployment attempts and having surgical backup available.^1,6^ In this case, the decision to extract the Aveir™ VR device and implant an Aveir™ AR device was made by an experienced leadless operator based on intraprocedural fluoroscopic and echocardiographic findings. ICE was used during the procedure to help with pacemaker positioning.

A potential limitation of this novel approach is the shorter projected battery longevity of the Aveir™ AR device compared with the Micra™ device.^6^ This consideration would be particularly important in younger patients or in those with a high anticipated pacing burden. However, this patient’s advanced age and anticipated pacing burden made mildly reduced device longevity acceptable.

This case underscores the need for flexibility in device selection and highlights the need to investigate the potential benefit of imaging-based pre-implantation RV sizing. As modular leadless systems continue to advance, there may be greater opportunities to tailor devices to individual anatomy, with smaller or dedicated pediatric options in future development. While not standard practice, the off-label use of alternative modules within the Aveir™ platform may be appropriate in select cases when supported by procedural evidence and operator experience.

## Conclusion

This case illustrates the successful off-label use of an Aveir™ AR LPM implanted in the RV to treat permanent AF with pauses in a nonagenarian patient with severe functional tricuspid regurgitation and a small RV cavity. In patients with small RV cavities, the larger profile of the Aveir™ VR may hinder secure fixation and result in suboptimal pacing thresholds and interaction with the TV. Our experience highlights how the modular design and cross-compatibility of Aveir™ AR system components can facilitate personalized solutions in anatomically challenging scenarios. While further investigation is warranted, this approach broadens the clinical applicability of leadless pacing and emphasizes the importance of detailed anatomical assessment in device selection.

## Supporting information

Supplementary Video 1: Video 1 showing original AveirTM VR device interacting with the tricuspid apparatus

Supplementary Video 2: Video 2 demonstrating snaring of the AveirTM VR device using the retrieveable catheter.

Supplementary Video 3: Video 3 showing final result with stable AveirTM AR device in inferior septum of right ventricle.
